# Heart rate variability behavior in young men after short-term carotenoid-containing supplementation

**DOI:** 10.1016/j.heliyon.2023.e14102

**Published:** 2023-03-02

**Authors:** Hsien-Tsai Wu, Jian-Jung Chen

**Affiliations:** 1 Department of Electrical Engineering, National Dong Hwa University, No. 1, Section 2, Da Hsueh Rd., Hualien, 97401, Taiwan; 2 Department of Chinese Medicine, Taichung Tzu Chi Hospital , Buddhist Tzu Chi Medical Foundation, Taichung, 42743, Taiwan; 3 Institute of Medical Sciences, Tzu Chi University, Hualien, 97002, Taiwan

**Keywords:** Carotenoid-Containing Supplementation, Heart Rate Variability (HRV), Cardiovascular disease (CVD), analysis of variance (ANOVA), percussion entropy index (PEI)

## Abstract

**Background:**

Heart rate variability can reflect the risk of developing cardiovascular disease (CVD), while carotenoids are good for CVD prevention. However, the acute effect of short-term carotenoid-containing supplementation on heart rate variability in young men is unclear.

**Methods:**

Thirty young men between 20 and 29 years of age without personal or family history of cardiovascular diseases were randomly divided into control and experimental groups. The anthropometric data, physiological parameters, and serum biochemical data were acquired, which were without significant difference between the two groups, at the beginning of trial. The participants in the experimental group consumed one pack of compound nutritional supplements in the morning (e.g., 10 AM) and another pack at night (e.g., 7 PM) each day. Heart rate variability was measured again once a month. Repeated measures analysis of variance with Roy’s largest root test and Bonferroni post hoc test were applied for primary outcomes.

**Results:**

Repeated measures analysis of variance indicated a significant time interaction effect for the estimated marginal means of percussion entropy index scale (T1 versus T3, T1 versus T4, and T2 versus T4 with *p* = 0.009, 0.005, and 0.032, respectively). Roy’s largest root test indicates there were significant differences between the means of the index after the intervention between two groups only on T3 and T4 (*p* = 0.007, *η*^2^ = 0.232 and *p* = 0.028, *η*^2^ = 0.162, respectively).

**Conclusion:**

Short-term carotenoid-containing supplementation could help young men by increasing heart rate variability capacity compared to controls over three months.

## 1 Introduction

Cardiovascular disease (CVD) is a growing global health threat that affects not only high-income countries but also those with low income [1]. More importantly, significantly younger subjects who are at risk of developing CVD have been focused on in recent years [2], similarly to how elder subjects and their CVD risk was a subject of focus a decade ago. A previous study addressed young adults with type 2 diabetes and arterial stiffness with a poor cardiovascular risk profile, specifically risk factors related to the lower HRV and metabolic syndrome **[**3**].** Accordingly, the findings in our earlier study [4] suggested that the non-linear index of HRV (e.g., percussion entropy index, PEI) was effective in the early identification of young men at high atherosclerotic risk of CVD and thus an important protective factor. Moreover, a previous study found increased sympathetic activity (HRV dysfunction) associated with a higher risk of cardiovascular disease [5]. A number of similar studies [[3], [4], [5], [6]] reported that greater HRV was modestly associated with lower risk of CVD during lifetime. Therefore, in this study, we attempted to adopt a non-linear HRV index [4,7] to reflect the health status of the cardiovascular systems of young subjects with and without carotenoid-containing supplementation.

In addition to improving the different presently available treatment strategies, increasing attention has been paid to the development of preventive measures. That includes the identification of risk factors for CVD, the early detection of diseases as well as the use of prophylactic medications and nutritional supplements in clinical practice [8]. In contrast to prophylactic treatments that act directly on blood vessels, the effects of carotenoid-containing supplements are less well-defined [9]. The results of previous studies investigating the benefits of nutritional supplements in subjects at risk of CVD are varied [10]. While some studies demonstrated a positive impact of nutritional supplements on the cardiovascular system [[11], [12], [13]], no evidence of any impact was found in other reports [14]. On the other hand, although carotenoids (any of a class of mainly yellow, orange, or red-fat-soluble pigments) were known of much earlier, details of their synthesis and metabolic products were not elucidated until the 1960s. Many receptors and hormones may also be involved in the metabolism of carotenoids and need to be explored at molecular level [15]. In a previous review study [16], the authors discussed the current research being undertaken to increase carotenoid contents in plants and the benefits to human health. The pathophysiology of many chronic and acute conditions, especially of CVD, is explained by inflammation and oxidative stress. Numerous studies have independently confirmed that carotenoids possess antioxidant biological properties and are well known to be important for human health and CVD prevention [[17], [18], [19]].

Although the majority of young men are free from cardiovascular disease (CVD), this group of subjects is gradually acquiring more CVD risk factors, especially due to unhealthy lifestyle factors, such as poor dietary habits, low exercise, no nutritional supplementation, and lack of sleep [20]. The study in [21] concluded that alterations in autonomic balance are already present in young adults, and metabolic syndrome was associated with lower HRV based on the frequency domain linear HRV index in 1889 subjects aged 24–39 years. On the other hand, the aim of a recent study [22] was to determine the effectiveness of heart rate biofeedback training on HRV and blood pressure in individuals with a family history of CVD by investigating the relationship between HRV and blood pressure in young adults at risk for CVD. Recently, Pearson correlation analyses [23] showed that the increased levels of carotenoids and vitamins were positively correlated with higher HRV (based on frequency domain linear HRV index) in 1074 (aged 34–84) individuals. From the viewpoint of data analysis, that study focused on creating a first step towards a comprehensive approach to the impacts of short-term carotenoid-containing supplementation on HRV in young men. Specifically, one novel application of a non-linear HRV index as well as the repeated measures ANOVA method was performed to assess the effects. We hypothesized that short-term carotenoid-containing supplementation (good for reducing the risk of CVD) of young men, i.e., over a period of three months, could improve HRV, thereby reducing the risk of CVD. As such, the aim of the study was to investigate the effects of providing the carotenoid-containing supplementation during the trials on changes in the young men’s HRV over a three-month intervention program.

## 2 Materials and Methods

### 2.1 Study Population, Grouping, and Experimental procedure

#### 2.1.1 Study Protocol

This study adopted a modified air pressure sensing system (APSS) to obtain pressure signals from wrist for medical index computation (e.g., PEI for heart rate variability (HRV)) [7,24] over 16 minutes. Our aim was to investigate PEI with and without short-term carotenoid-containing supplementation in young men with regard to the different clinical effects as determined under repeated measures ANOVA.

#### 2.1.2 Random Grouping and Intervention

##### A Inclusion criteria

Among July 14, 2017 to June 13, 2018 and November 2011 and May 2012, a total of 35 young-aged males aged between 20 and 29 were recruited for investigation (5 subjects quit before the end of the experiment, i.e., control group, n = 20, and experimental group, n = 10, all HbA1c ＜ 6.5%) in a randomized controlled trial (i.e., the complete randomization was typically performed by a computer program). All of the age-controlled healthy young subjects had no personal or family history of cardiovascular disease.

##### B Exclusion criteria

Vegetarians and those who had previously consumed daily nutritional supplements with in the last 6 months were excluded in this study. More importantly, subjects without successful measurements of heart rate variability were also excluded from the study.

The participants would receive 60 packs of compound nutritional supplements each month. For the current study, the participants in the experimental group consumed one pack of compound nutritional supplements in the morning (e.g., 10 AM) and another pack at night (e.g., 7 PM) each day (double-check by telephone). The reference compounds are commercially available (LifePak, PHARMANEX ®, Utah, USA). Each pack contains one vitamin tablet (667 mg), one phytonutrient tablet, and two mineral tablets. Each commercially available pack of compound carotenoid-containing supplements contained the following: β-carotene (3 mg), folate (300 μg), lutein (20 mg), lycopene (15 mg), vitamin K1 (40 μg), vitamin E (134 mg), vitamin D3 (5 μg), vitamin C (500 mg), vitamin B12 (30 μg), vitamin B6 (10 mg), vitamin B1 (7.5 mg), vitamin B2 (4.25 mg), and vitamin A (1500 mg). On the other hand, the participants in the control group did not take the above carotenoid-containing supplements. Moreover, all participants were asked not to change their lifestyle in the trials. The dose of micronutrients in the study could supply a comprehensive blend of nutrients to support a healthy cardiovascular system [25]. In addition, the reference compounds for LifePak are not all the same for the young males, young females, and elder people in the study. To simplify research, the current study is a better way to focus on young males.

#### 2.1.3 Experimental procedure

On the day of first examination (T1), the participants were required to fast for at least 8 hours and were taken to the outpatient clinic department in Hualien Hospital for medical assessment and blood samples, including determination of high-density lipoprotein (HDL), low-density lipoprotein (LDL), triglyceride, total cholesterol, fasting blood sugar concentration, and glycosylated hemoglobin (HbA1c) concentrations. Then, participants waited outside the clinic until the doctor’s assessment. When the doctor’s assessment was done, the subjects were taken to a health clinic for physiological data measurements (e.g., age, body weight, height, waist circumference, and blood pressure), completion of the questionnaire, and computation of HRV. All the physiological data measurements were taken in a temperature controlled room (26 ± 1^o^C). Subsequently, the subjects were asked to take a five minute rest, then two sets of refined APSS pressure cuffs (i.e., a wrist cuff and an upper arm cuff) were attached to the right arm for measurement of PEI over 16 minutes. In addition, PEI measures were conducted again after one month (T2), two months (T3), and three months (T4). The Institutional Review Board (IRB) of Hualien Hospital and Taichung Tzu Chi Hospital approved, and the data was used this study.

### 2.2 Percussion entropy index for HRV assessment

For two time series (i.e., peak-to-peak intervals (PPI) and waveform amplitudes (WA) series of 9 min WPP in reactive hyperemia phase) of length N= 700, the modified percussion entropy index is computed with the following three steps algorithm:

1. A binary transformation of PPI and WA is used to obtain a = {a_1_, a_2_, ..., a_N_} and b = {b_1_, b_2_, ..., b_N_}, respectively in the following equations (1), (2):

(1) $$a_{i}=\left\{ \begin{array}{c} 0,PPI\left(i+1\right)\leq PPI\left(i\right)\\ 1,PPI\left(i+1\right)>PPI\left(i\right) \end{array}\right.\text{;}$$

(2) $$b_{i}=\left\{ \begin{array}{c} 0,WA\left(i+1\right)\leq WA\left(i\right)\\ 1,WA\left(i+1\right)>WA\left(i\right) \end{array}\right.\text{;}$$

2. The percussion rate for each scale factor s is obtained as:

(3) $${\mathrm{PR}}_{s}^{m}=\frac{1}{\left(n-m-s+1\right)}\sum_{i=1}^{n-m-s+1}count\left(i\right)\text{,}$$

where m is the embedded dimension vectors and count(i) represents the match number between a(i) = {a_i_, a_i+1_, ..., a_i+m-1_} and b(i+s) = {b_i+s_, b_i+s+1_, ..., b_i+s+m-1_};

3. PEI is calculated as:

(4) $$PEI\left(m,S\right)=\ln\left[\frac{\sum_{s=1}^{Si}{\mathrm{PR}}_{s}^{m}}{\sum_{s=1}^{Si}{\mathrm{PR}}_{s}^{m+1}}\right],\ln\;is\;natural\;logarithmic\;operator\text{,}$$

(5) $${where\;\mathrm{PR}}_{s}^{m+1}=\frac{1}{\left(n-m-s+2\right)}\sum_{i=1}^{n-m-s+2}count\left(i\right)\text{,}$$

where equations (3), (4), (5) show m = 2, and s = 1 for healthy young men in this study. ${The\;percussion\;rates\;(\mathrm{PR}}_{s=1}^{2}$ and ${\mathrm{PR}}_{s=1}^{3}$) were with different lengths of fluctuation vectors 2 and 3, respectively. The percussion count in equation (3) increased one when the two compared patterns of fluctuation were identical. A high percussion rate represents high similarity in the pattern of fluctuation and indicates the subject is healthier. The percussion count number increased 1 when the two compared patterns of fluctuation were identical. The high PEI values in equation (4) represented the test subject with more healthy, as addressed in [7,24].

### 2.3 Statistical Analysis

The statistical software package, Version 14.0 (SPSS Inc., Chicago, IL, USA) was used to analyze the data. Descriptive statistics (means, standard deviations, and frequencies) were used to describe the young male subjects. The values in Table 1 are represented as the means ± standard deviation (SD), and a single-sample Kolmogorov–Smirnov test was adopted for testing the normality of the distribution followed by non-parametric tests (i.e., Mann-Whitney-Wilcoxon test). Changes in dependent variable (i.e., PEIs for four measurements) and between-subject factors (i.e., control group vs. experimental group) from baseline to post-measures were assessed using repeated measures analysis of variance (ANOVA) to determine “time” and “time-by-group” differences using Roy’s largest root test after parametric tests checking [26]. To conduct multiple comparisons, Bonferroni post hoc test for unequal sample sizes was adopted. Results were considered significant at p < 0.05. Finally, two illustrated plots for estimated marginal means of PEI versus group and time were shown to allow an easy understanding of the different effects for the experimental group compared to the control group.

**Table 1:** General characteristics (mean, standard deviation, and frequency) of the participants.

Parameter	Control Group	Experimental Group	*p* Values
	Mean ± SD or N (%)	Mean ± SD or N (%)	
**Number of young men**	20 (66.7%)	10 (33.3%)	N/A
**Age, year**	23.96 ± 2.46	23.28 ± 3.30	0.210
**Body height, cm**	173.08 ± 5.47	174.89 ± 6.28	0.355
**Body weight, kg**	68.73 ± 9.62	70.75 ± 13.18	0.328
**WC, cm**	78.81 ± 8.17	79.67 ± 10.23	0.510
**BMI, kg/m2**	24.82 ± 3.88	26.91 ± 4.21	0.277
**SBP, mmHg**	119.08 ± 13.74	118.83 ± 12.64	0.689
**DBP, mmHg**	77.65 ± 7.84	76.95 ± 9.14	0.798
**HDL, mg/dL**	47.52 ± 7.51	42.83 ± 6.91	0.182
**LDL, mg/dL**	89.68 ± 17.56	97.82 ± 23.46	0.179
**TC, mg/dL**	154.55 ± 19.39	160.84 ± 21.67	0.713
**TG, mg/dL**	91.48 ± 24.91	94.17 ± 31.32	0.312
**FBS, mg/dL**	92.48 ± 4.95	94.56 ± 8.38	0.457
**HbA1c, %**	5.34± 0.27	5.41 ± 0.38	0.513

The final number of test subjects was 30. WC, waist circumference; BMI, body mass index; SBP, systolic blood pressure; DBP, diastolic blood pressure; HDL, high-density lipoprotein; LDL, low-density lipoprotein cholesterol; FBS, fasting blood sugar; HbA1c, glycosylated hemoglobin. The *p* values of parameters larger than 0.05 are regarded as not statistically significant between the two groups for the nonparametric Mann-Whitney-Wilcoxon test. The *p*-values of control group versus experimental group for the normality test were listed: Age(0.021, 0.008), Body height(0.138, 0.018), Body weight(0.354, 0.020), WC(0.316, 0.019), BMI(0.409, 0.027), SBP(0.390, 0.031), DBP(0.198, 0.058), HDL(0.154, 0.037), LDL(0.179, 0.018), TC(0.127, 0.025), TG(0.843, 0.042), FBS(0.252, 0.047), and HbA1c(0.047, 0.008).

## 3 Results

It is an important issue for young males (randomly grouped but with a small size in the study) to take care of their health, and it also depends on them understanding how to assess their cardiovascular system and knowing what steps are needed to improve their health and substantially delay CVD. Repeated measures analysis of variance (ANOVA) was adopted to assess the health status of the cardiovascular systems of young males after short-term carotenoid-containing supplementation.

### 3.1 Baseline General Characteristics of Randomly Grouped Subjects

There were no statistically significant differences in many of the investigated variables (e.g., the demographic data (age), anthropometric data (body height, body weight, waist circumference), physiological parameters (systolic blood pressure and diastolic blood pressure), and serum biochemical data (high-density lipoprotein, low-density lipoprotein, cholesterol, triglyceride, fasting blood sugar, glycosylated hemoglobin)) between control and experimental groups at the baseline of the intervention (before taking carotenoid-containing supplementation every day) (all *p* > 0.05) (Table 1).

### *3.2* *Repeated Measures* ANOVA

The Levene's test of equality of error variance was performed for the null hypothesis that the error variance of the dependent variable is equal across groups in the study.

#### ● Repeated measures ANOVA and Bonferroni post hoc test

All of the *p*-values of PEIs for four measurements for control group and experimental group for the normality test were larger than 0.05 in Table 2. Therefore, all the PEIs for four measurements are normally distributed set of data. The repeated measures ANOVA indicates a significant TIME interaction effect for the estimated marginal means of PEI scale (T1 versus T3, T1 versus T4, T2 versus T4 with *p* = 0.009, 0.005, and 0.032, respectively) (Table 2). Base on the fact that PEIs for four measurements are normally distributed, the appropriate steps for interpreting the SPSS output with a repeated-measures ANOVA and Bonferroni post hoc tests were adopted in Table 3, Table 4. Table 3 also addressed tests of between-subjects effects (F = 5.467, *p* = 0.027, η^2^ = 0.163). Roy’s largest root test indicated a significant difference between means of PEI after the intervention between two groups only for T3 and T4 (*p* = 0.007, η^2^ = 0.232; *p* = 0.028, η^2^ = 0.162, respectively). None of the other scales showed any TIME or TIME × GROUP interaction effects (Table 4).

**Table 2:** Pairwise comparisons based on estimated marginal means of PEI between two different measurements with Bonferroni adjustment for multiple comparisons.

(I) time	(J) time	Mean Diff. (I-J)	Std. Error	*p* Values	95% CI for Difference^a^	
					Lower Bound	Upper Bound
T1	T2	-2.458	0.872	0.052	-4.932	0.017
	T3	-3.195^∗^	0.908	**0.009**	-5.773	-0.617
	T4	-5.613^∗^	1.505	**0.005**	-9.884	-1.341
T2	T1	2.458	0.872	0.052	-0.017	4.932
	T3	-0.738	0.829	1.000	-3.092	1.617
	T4	-3.155^∗^	1.045	**0.032**	-6.122	-0.188
T3	T1	3.195^∗^	0.908	**0.009**	0.617	5.773
	T2	0.738	0.829	1.000	-1.617	3.092
	T4	-2.417	1.049	0.173	-5.396	0.561
T4	T1	5.613^∗^	1.505	**0.005**	1.341	9.884
	T2	3.155^∗^	1.045	**0.032**	0.188	6.122
	T3	2.417	1.049	0.173	-0.561	5.396

All of the p-values of PEIs for four measurements for control group and experimental group for the normality test were larger than 0.05 using Kolmogorov–Smirnov test.

Adjustment for multiple comparisons: Bonferroni. The mean difference is significant at the 0.05 level.

CI: confidence interval.

**Table 3:** Tests of between-subject effects.

Source	Type III Sum of Squares	df	Mean Square	F	*p* Values	Partial Eta Squared (*η*^2^)
	385970.522	1	385970.522	16311.522	0.000	0.998
Group	129.360	1	129.360	5.467	0.027	0.163
Error	662.548	28	23.662			

The factor “Group” means experimental group versus control group (reference).

**Table 4:** Parameter estimates of different dependent variables (T1, T2, T3, and T4) for the experimental group (n = 10) versus the control group (n = 20).

Dependent Variable	Parameter	B	Std. Error	*p* Values
T1	Intercept	56.800	1.613	0.000
	Group = 2	-1.075	1.976	0.591
T2	Intercept	60.750	1.007	0.000
	Group = 2	1.910	1.234	0.133
T3	Intercept	62.500	1.104	0.000
	Group = 2	3.935	1.352	**0.007**
T4	Intercept	64.970	1.420	0.000
	Group = 2	4.040	1.740	**0.028**

B: regression coefficient; Group = 2; i.e., experimental group, control group (Group = 1) is regarded as reference.

#### The changes in estimated marginal means of PEI with “time” and “group” views

**Figure 1:**
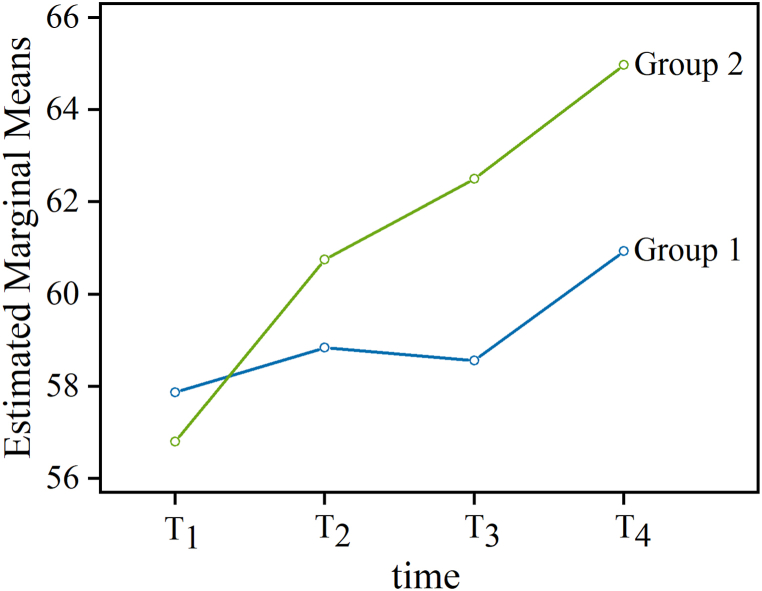
The changes in estimated marginal means of PEI within the four time periods for the experimental group (n = 10) versus the control group (n = 20). The estimated marginal means of PEI for the experimental group obviously increased under longer intervention times (e.g., PEI: 56%∼65%). On the other hand, the estimated marginal means of PEI for the control group did not change much (e.g., PEI: 58%∼61%).

**Figure 2:**
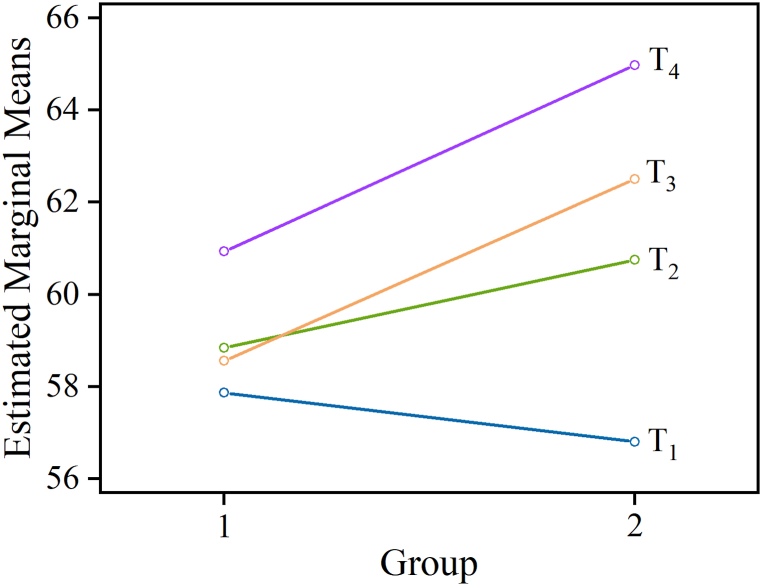
Distribution of the estimated marginal means of PEI scale scores for the experimental and control groups during the four measurement periods ((T1, T2, T3, and T4). It is obvious that the experimental group presented smaller estimated marginal means of PEI than the control group at the beginning of the trial. Subsequently, the experimental group presented larger estimated marginal means of PEI than the control group in the T2, T3, and T4 measurement periods.

The changes in estimated marginal means of PEI with “time” and “group” views. For the control group, all scales with the exception of one decreased in their mean values from T1 to T4. As illustrated in Figure 1, although the estimated marginal means of PEI of the experimental were smaller than the control group at T1 measurement, the values of the experimental group increased significantly more than the control group from T2 to T4, resulting in the experimental group having substantially higher scores than the control group after intervention (i.e., T2, T3, and T4). From another perspective, the estimated marginal means of PEI for the experimental and control groups for the four measurement periods ((T1, T2, T3, and T4) are shown in Figure 2. At the beginning of the trial, the estimated marginal means of PEI of the control group were larger than the experimental group. However, the estimated marginal means of PEI of the experimental group were larger than the control group after intervention (i.e., T2, T3, and T4).

## 4 Discussion

As a pilot study that may be enlarged to more people evaluating the effect on a long term, the primary objective of this study was to investigate the effects of providing short-term carotenoid-containing supplementation on PEI in equation (4) changes in young men over a three-month intervention program. The effectiveness of an intervention is usually evaluated through the changes in the mean values of the experimental group, representing progress or regress of the HRV variable. In the present study, the hypothesis was confirmed only for one variable. The daily carotenoid-containing supplementation improved test subjects’ estimated marginal means of PEI in the experimental group (F = 5.467, *p* = 0.027, η^2^ = 0.163) (Table 3). Similar to the current study, a previous study showed that increased levels of carotenoids and vitamins were positively correlated with higher HRV [27]. While the control group (the group without taking carotenoid-containing supplementation every day) had larger estimated marginal means of PEI than those of the experimental group in the first month (T1 period), these results were not statistically significant (Figure 1, Figure 2). More importantly, a significant time interaction effect for estimated marginal means of PEI scale shown was by repeated measures ANOVA (T1 versus T3, T1 versus T4, T2 versus T4 with *p* = 0.009, 0.005, and, respectively) (Table 2). In addition, a significant difference between the means of estimated marginal means of PEI after the intervention between two groups only on T3 and T4 was also indicated by Roy’s largest root test (*p* = 0.007, η2 = 0.232 and *p* = 0.028, η2 = 0.162, respectively) (Table 4). In a recent study [23], the authors found that blood concentrations of antioxidant micronutrients, carotenoids, and vitamins were associated with beneficial changes according to the values of frequency domain linear HRV index in a cross-sectional analysis. In addition, study in [28] was designed to evaluate the consumer-perceived efficacy of an oral supplement containing a mix of tomato carotenoids and oil-soluble vitamins in improving skin appearance after 12 weeks of supplement use in 60 females, aged 35 to 55 years. Therefore, in this study, we attempted to adopt a non-linear HRV index to reflect the health status of the cardiovascular systems of young males (not female) with and without carotenoid-containing supplementation. With all considered, it is not likely that normal hormone cycles/fluctuations in a cross-sectional analysis are going to have a profound effect on young females. In addition, the reference compounds (LifePak, PHARMANEX®, Utah, USA) in the study are not all the same for young male, young female and elder person in the study. For simplify research, the current study is a better way to focus on young male. Finally, a randomized, controlled pilot trial was implemented in this study, and short-term carotenoid-containing supplementation helped young men to increase their HRV capacity compared to the control group over three months.

A recent study [29] addressed the effects of body composition on the cardiovascular system, especially focusing on HRV and vascular endothelial function. In the study by Weggen et al., after acute antioxidant supplementation, lower vascular or autonomic function was found in the recipients undergoing the supplementation regime (vitamins C and E; α-lipoic acid) compared to in the healthy controls, potentially implicating oxidative stress as a contributor to this blunted vascular or autonomic function [30]. A number of integrative studies have demonstrated decreases in blood pressure, cholesterol, blood glucose, and measures of stress, which all impact cardiovascular morbidity and mortality [31], in response to short-term carotenoid-containing supplementation; however, its application in young men is novel, and the impacts on HRV have not been previously reported. The aim of this study was to demonstrate that short-term carotenoid-containing supplementation improves HRV in young men, thus serving as further encouragement to young adults to take up healthier lifestyles. Nutritional supplementation was identified in a recent study as one of the important factors determining success in attempts to optimize public health [7,32]. Therefore, the promotion of healthy lifestyles and the prevention of ill health are fundamental to public health. Hence, young men need to understand that their cardiovascular system can benefit from carotenoid-containing supplementation and should be encouraged to live healthier lifestyles [33].

Some strengths and limitations of this study are worth noting. The most important strength is the experimental nature of the study, including the use of a new non-linear cross-entropy index of HRV. The study demonstrated that short-term carotenoid-containing supplementation could help young men by increasing heart rate variability capacity compared to controls over three months. However, this work is still preliminary and has some limitations, like the small sample size, the long term effect of diet supplementation, and the restriction only to male. Furthermore, to our best knowledge, this is the first time this type of research on short-term carotenoid-containing supplementation effects on HRV has been conducted on young men in Taiwan. Second, although it is well accepted that dietary intake and physical activity are significant factors affecting cardiovascular heath for young males, details on the dietary intake and/or participants’ exercise habits were not available. Third, the effects of the carotenoid-containing supplement on females were not studied due to the design of the present study intentionally avoiding confounding factors of this gender. Finally, the methods adopted in the present study for the assessment of HRV are not commonly used and have not been standardized in the international literature. More data are needed to strengthen their uses in this clinical setting.

## 5 Conclusions

The results of this study show that a short-term carotenoid-containing supplementation program had an impact on HRV regulation in young men. The findings suggest that young males who have not only high daily intakes of vegetables and fruit but also carotenoid-containing supplementation could benefit from good impacts on HRV regulation and the cardiovascular system.

### Author contribution statement

Jian-Jung Chen: Conceived and designed the experiments; Performed the experiments; Contributed reagents, materials, analysis tools or data; Wrote the paper.

Hsien-Tsai Wu: Performed the experiments; Analyzed and interpreted the data; Contributed reagents, materials, analysis tools or data; Wrote the paper.

### Funding statement

This research did not receive any specific grant from funding agencies in the public, commercial, or not-for-profit sectors.

### Data availability statement

Data will be made available on request.

### Declaration of interest’s statement

The authors declare no competing interests.

## Institutional Review Board Statement

All subjects were asked to submit written consent during the experimental procedure. This nonrandomized experimental study **(protocol no.: TTCRD106-18)** was conducted from July 14, 2017 to June 13, 2018 in Taichung Tzu Chi Hospital, Taiwan, according to the protocol approved by the Research Ethics Committee of Taichung Tzu Chi Hospital. The study protocol was also approved by the Institutional Review Board of Hualien Hospital, Taiwan (protocol no. 98-06-02 and date of approval (16 July 2011)) and procedures were in accordance with the ethical standards of the responsible committee on human experimentation and with the Helsinki declaration of 1975, as revised in 1983.

## Informed Consent Statement

Informed consent was obtained from all subjects involved in the study.

## Code availability

The PEI computation programs are accessible upon demand from the authors
