# Supplementary material for: Heart rate variability behavior in young men after short-term carotenoid-containing supplementation
Source: Heliyon. 2023 Mar 2;9(3):e14102. doi: 10.1016/j.heliyon.2023.e14102 (PMC10009683; doi:10.1016/j.heliyon.2023.e14102)
Supplement: Supplementary file 1 [file mmc1.docx]

**Questionnaire**

| \| **Article Publishing Charge**  *Heliyon* is a fully Open Access journal. The journal’s costs are covered solely by author publication charges. There are no subscription fees for our readers, or page and figure charges for our authors. Accordingly, all authors of accepted articles will receive an invoice charging the article publication fee of $1,950 USD (plus VAT and local taxes where applicable).  *Heliyon* has a small budget for reducing Open Access charges for authors in developing countries and others in genuine financial hardship. We will grant 100% and 50% discounts on the article publication fee for requests from authors from [Research4Life group A and group B countries](https://www.research4life.org/access/eligibility/), respectively. Please note that funds for other reductions are limited, however, as the journal would not be sustainable without income from author charges to cover its cost, we do encourage authors to consult with their co-authors, institutional libraries and departments in the cases where there is a gap to cover the publication fee. Authors who have received a special offer or a personal invitation should add a short explanatory note to their cover letter.  For any questions around open access, including the application for a discount on the basis of financial hardship, please contact our open access support team through this [webform](https://service.elsevier.com/app/contact/supporthub/publishing/) using the options author/ Open Access and the relevant subject. Please note that requests in the cover letter or other parts in the manuscript are not considered and that the application for support must be submitted through the form outlined above as we are separating the editorial considerations from the financial aspects of the journal. Requests from authors with funding outlined [here](https://www.elsevier.com/about/open-science/open-access/agreements) cannot be considered. \| \| \| --- \| --- \| \| Answer Required: \| \| Please select a response \| \| --- \| \| Yes, I have read and understood the information regarding open access and understand that I have to pay an article publication fee upon acceptance of my manuscript. \| \| | | | | |
| --- | --- | --- | --- | --- | --- | --- | --- | --- | --- | --- |
|  | |  |  | |
| \| **Publication ethics** Please confirm that you have reviewed our guidelines for [Ethics in Publishing](https://www.elsevier.com/about/policies/publishing-ethics). \| \| \| --- \| --- \| \| Answer Required: \| \| Please select a response \| \| --- \| \| I confirm \| \| | | | | |
|  |  | | |  |

| \| **Category**  Select the Category related to your submission from the drop down menu: \| \| \| --- \| --- \| \| Answer Required: \|  \| | | |
| --- | --- | --- | --- | --- | --- | --- |
|  | \| \| Select the Section Category related to your submission from the drop down menu: \| \| \| --- \| --- \| \| Answer Required: \|  \| \| \| \| \| --- \| --- \| --- \| --- \| --- \| --- \| --- \| \|  \|  \|  \| |  |
| \| **Clinical Study** Does your study include a clinical study? \| \| \| --- \| --- \| \| Answer Required: \|  \| | | |
|  | \| \| Is your study an (interventional) clinical trial or an observational study? \| \| \| --- \| --- \| \| Answer Required: \|  \| \| \| \| \| --- \| --- \| --- \| --- \| --- \| --- \| --- \| \|  \|  \|  \| |  |

| \| **Animal Experiments** Does your research involve experimentation on animals? \| \| \| --- \| --- \| \| Answer Required: \|  \| | | | | |
| --- | --- | --- | --- | --- | --- | --- | --- | --- |
|  | |  |  | |
| \| **Human Subjects** Does your study include human subjects? \| \| \| --- \| --- \| \| Answer Required: \|  \| | | | | |
|  | \| \| Please provide the name of the ethical committee approving these experiments. \| \| \| --- \| --- \| \| Answer Required: \| \| Character Count: 24 \| \| \| \| --- \| --- \| --- \| \|  \| Limit 200 characters \|  \| \| \| \| \| \| \| \| --- \| --- \| --- \| --- \| --- \| --- \| --- \| --- \| --- \| --- \| --- \| --- \| --- \| --- \| --- \| \|  \| \|  \|  \| \| \| \| Please confirm authors’ compliance with all relevant ethical regulations. \| \| \| --- \| --- \| \| Answer Required: \|  \| \| \| \| \| \| \|  \| \| \| Please confirm that written consent has been obtained from all patients/participants. \| \| \| --- \| --- \| \| Answer Required: \|  \| \| \| \| \| --- \| --- \| --- \| --- \| --- \| --- \| --- \| \|  \|  \|  \| \| \| \|  \| | | |  |

| \| **Data Availability**  Sharing research data helps other researchers evaluate your findings, build on your work and to increase trust in your article. We encourage all our authors to make as much of their data publicly available as reasonably possible. Please note that your response to the following questions regarding the public data availability and the reasons for potentially not making data available will be available alongside your article upon publication.  Has data associated with your study been deposited into a publicly available repository? \| \| \| --- \| --- \| \| Answer Required: \|  \| | | | | |
| --- | --- | --- | --- | --- | --- | --- | --- | --- |
|  | \| \| Please select why. Please note that this statement will be available alongside your article upon publication. \| \| \| --- \| --- \| \| Answer Required: \|  \| \| \| \| \| --- \| --- \| --- \| --- \| --- \| --- \| --- \| \|  \|  \|  \| | | |  |
| \| **Data in Brief**  Does your submission include Data in Brief (optional)? If so, please upload all Data in Brief files *(completed Word template and any relevant data files)* as a single zip file, and select "Data in Brief" as File Type. \| \| \| --- \| --- \| \| Answer Required: \| \| Please select a response \| \| --- \| \| My submission contains a Data in Brief zip file. \| \| My submission does not include Data in Brief. \| \| | | | | |
|  | |  |  | |

| \| **Declaration of interests** Transparency is essential for a reader’s trust in the scientific process and for the credibility of published articles. At Cell Press, we feel that disclosure of competing interests is a critical aspect of transparency. Therefore, we require that all authors disclose any financial or other interests related to the submitted work that (1) could affect or have the perception of affecting the author’s objectivity, or (2) could influence or have the perception of influencing the content of the article. For more information please refer to our [ethics page](https://www.cell.com/heliyon/ethics).  **Institutional Affiliations** We require that you list the current institutional affiliations of all authors, including academic, corporate, and industrial, on the title page of the manuscript. Please select one of the following: \| \| \| --- \| --- \| \| Answer Required: \| \| Please select a response \| \| --- \| \| All affiliations for all co-authors are listed on the title page of the manuscript. \| \| I or other authors have additional affiliations that we have noted in the “Declaration of Interests” free text section in this questionnaire below. \| \| | | |
| --- | --- | --- | --- | --- | --- | --- | --- | --- | --- |
|  |  |  |
| \| **Funding sources** We require that you disclose all funding sources for the research described in this work. This information will be published alongside your work. Please confirm the following: \| \| \| --- \| --- \| \| Answer Required: \| \| Please select a response \| \| --- \| \| All funding sources for this study are listed in the “Funding Information” section of the Manuscript Data step of the submission process (next page). \| \| | | |
|  |  |  |

| \| **Competing Financial Interests** We require that authors disclose any financial interests and any such interests of immediate family members, including financial holdings, professional affiliations, advisory positions, board memberships, receipt of consulting fees etc., that:   1. could affect or have the perception of affecting the author’s objectivity, or 2. could influence or have the perception of influencing the content of the article.   Please select one of the following: \| \| \| --- \| --- \| \| Answer Required: \| \| Please select a response \| \| --- \| \| We, the authors and our immediate family members, have no financial interests to declare. \| \| We, the authors, have noted any financial interests in the “Declaration of Interests” free text section in this questionnaire below, and we have noted interests of our immediate family members. \| \| | | | | |
| --- | --- | --- | --- | --- | --- | --- | --- | --- | --- | --- | --- |
|  | |  |  | |
| \| **Advisory/Management and Consulting Positions** We require that authors disclose any position, be it a member of a Board or Advisory Committee or a paid consultant, that they have been involved with that is related to this study. We also require that members of our journal editorial teams or advisory boards disclose their position when publishing in the journal. Please select one of the following : \| \| \| --- \| --- \| \| Answer Required: \| \| Please select a response \| \| --- \| \| We, the authors and our immediate family members, have no positions to declare and are not members of the journal’s editorial teams or advisory boards. \| \| The authors and/or their immediate family members have management/advisory or consulting relationships noted in the “Declaration of Interests” free text section in this questionnaire below. \| \| | | | | |
|  |  | | |  |

| \| **Patents** We require that you disclose any patents related to this work by any of the authors or their institutions. Please select one of the following : \| \| \| --- \| --- \| \| Answer Required: \| \| Please select a response \| \| --- \| \| We, the authors and our immediate family members, have no related patents to declare. \| \| We, the authors have a patent related to this work, which is noted in the “Declaration of Interests” free text section in this questionnaire below, and we have noted the patents of immediate family members. \| \| | | | | |
| --- | --- | --- | --- | --- | --- | --- | --- | --- | --- | --- | --- |
|  | |  |  | |
| \| Please insert any “Declaration of Interests” statement in this space. If no authors have a competing interest, please insert the text 'The authors declare no competing interests'. This text will be published alongside your article. \| \| \| --- \| --- \| \| Answer Required: \| \| Character Count: 42 \| \| \| \| --- \| --- \| --- \| \|  \| Limit 20000 characters \|  \| \| | | | | |
|  |  | | |  |

| \| **Heliyon Null Hypothesis** Heliyon is launching a Null Hypothesis collection, designed to highlight valuable research presenting results which do not support the proposed hypothesis. Does your study contain results which do not support your hypothesis, sometimes called “negative” results? Your answer to this question will not affect the decision process for your manuscript, but will ensure your article, if published, will receive adequate visibility to underscore the importance of these studies for the scientific community. \| \| \| --- \| --- \| \| Answer Required: \| \| Please select a response \| \| --- \| \| Yes, my manuscript contains “negative” or “inconclusive” results. \| \| No, my manuscript does not contain “negative” or “inconclusive” results. \| \| | | | | |
| --- | --- | --- | --- | --- | --- | --- | --- | --- | --- | --- | --- |
|  |  | | |  |
| \| We encourage authors to share their Twitter usernames for all authors. These will be used to promote the article on Twitter should your manuscript be accepted. \| \| \| --- \| --- \| \|  \| \| Character Count: 0 \| \| \| \| --- \| --- \| --- \| \|  \| Limit 200 characters \|  \| \| | | | | |
|  | |  |  | |

| \| **Special Issue**  Is your manuscript being submitted to a special issue? \| \| \| --- \| --- \| \| Answer Required: \|  \| | | | | |
| --- | --- | --- | --- | --- | --- | --- | --- | --- |
|  | |  |  | |
| \| **Double anonymized peer review**  Heliyon offers the option of double anonymized peer review. Papers reviewed under this pilot program will not display author names to reviewers through the submission system, nor will author names be used in our communication with reviewers.  Please ensure that any reference to you as authors is removed from your manuscript file and that the authorship list is included in your cover letter only. Ensuring that all identifying details are removed from other submission files is responsibility and you can find more information can be found on our Guide for Authors page.  When your paper is sent for external peer review, should peer reviewers be able to see you and your co-authors' identities? \| \| \| --- \| --- \| \| Answer Required: \| \| Please select a response \| \| --- \| \| Yes, I request standard single anonymized peer review for my manuscript. \| \| No, I request double anonymized peer review for my manuscript. I have removed all identifying details from all submission files and only included my authorship list in in the cover letter. If you have not yet prepared your manuscript and auxiliary files in this way, please return to the Attach Files stage and replace the versions uploaded so far with updated ones. \| \|  \| \| | | | | |
|  | \| \| **Free Preprint Service**  Do you want to share your research early as a preprint? Preprints allow for open access to and citations of your research prior to publication.  Heliyon offers a free service to post your paper in a journal-branded [First Look](https://www.ssrn.com/index.cfm/en/heliyon/) space on SSRN, an open access research repository, when your paper enters peer review. Once on SSRN, your paper will benefit from early registration with a DOI and early dissemination that facilitates collaboration and early citations. It will be available free to read regardless of the publication decision made by the journal. This will have no effect on the editorial process or outcome with the journal. Please consult the [SSRN Terms of Use](https://www.ssrn.com/index.cfm/en/terms-of-use/) and [FAQs](https://service.elsevier.com/app/answers/detail/a_id/34422/supporthub/ssrn/kw/First+Look/). \| \| \| --- \| --- \| \| Answer Required: \| \| Please select a response \| \| --- \| \| YES, I want to share my research early and openly as a preprint. \| \| NO, I don’t want to share my research early and openly as a preprint. \| \| \| \| \| \| --- \| --- \| --- \| --- \| --- \| --- \| --- \| --- \| --- \| --- \| \|  \|  \|  \| | | |  |

Back
